# Dynamic changes of HPV genotypes in a tourism-influenced populated area, Northeastern Iran

**DOI:** 10.1371/journal.pone.0356563

**Published:** 2026-09-03

**Authors:** Afsane Fadaee, Seyed Sajjad Alavi-Kakhki, Samira Basharkhah, Seyed Ali Akbar Shamsian, Zahra Shadman, Narges Valizadeh, Samira Amin Poustchi Tousi, Mohammad Arefi, Fatemeh Sadat Mohammadi, Sanaz Ahmadi Ghezeldasht, Arman Mosavat, Seyed Abdolrahim Rezaee

**Affiliations:** 1 Immunology Research Center, Inflammation and Inflammatory Diseases Division, Mashhad University of Medical Sciences, Mashhad, Iran; 2 Student Research Committee, Mashhad University of Medical Sciences, Mashhad, Iran; 3 Department of Mycology and Parasitology, Faculty of Medicine, Mashhad University of Medical Sciences, Mashhad, Iran; 4 Saveh School of Medical Sciences, Saveh, Iran; 5 Blood Borne Infections Research Center, Academic Center for Education, Culture and Research (ACECR), Razavi Khorasan, Mashhad, Iran; Jordan University of Science and Technology, JORDAN

## Abstract

Human papillomavirus (HPV) comprises more than 230 genotypes; its oncogenic potential is a major concern, particularly in tourism-influenced regions. The present study was conducted to assess HPV prevalence and temporal changes in genotype distribution after an eight-year interval in subjects who were suspected of sexually transmitted infections (STIs). Professional physicians referred samples from 2,673 subjects to the main molecular diagnostic labs in North, Razavi, and South Khorasan provinces, as well as from a diverse group of tourists. The findings showed that HPV was detected in 1,185 (55.86%) females and 400 (72.46%) males (*p* = 0.01). HPV infection was most frequent in the 21–30 and 31–40 year-old groups, at 35.6% and 39.7%, respectively. Most cases clustered around five to six genotypes. The six most frequent genotypes included HPV-6 (56.62%; 77/136), HPV-16 (40.44%; 55/136), HPV-54 (36.03%; 49/136), HPV-43 (29.41%; 40/136), HPV-51 (29.41%; 40/136), and HPV-52 (28.68%; 39/136). Importantly, three prevalent genotypes (16, 51 and 52) are classified as HR-HPV. Compared with eight years ago, the percentage of HPV prevalence strongly increased by more than 28%, primarily in genital discharge samples. The most notable change (0.4% *vs* 5.1%) occurred in the proportion of individuals with more than four genotypes, *i.e.,* currently 11.7 times higher than eight years ago. The greatest shift in genotype prevalence has occurred between HPV-16 and HPV-51 over the past few years. Moreover, compared to the previous study, minimal changes (0.90%) in prevalence and genotypes have been observed in South Khorasan, the highest in North and Razavi Khorasan provinces (14.5%) and also in tourist groups (3.2%). Therefore, the impact of transient populations and more likely changes in socioeconomic and cultural status in these areas, along with the progressive trends in HPV infection, should prompt health authorities to focus more on establishing HPV diagnostic facilities, screening, vaccination, and increasing public knowledge.

## Introduction

Human papillomavirus (HPV) is a double-stranded DNA virus belonging to the *Papillomaviridae* family, with a genomic length of 7.2–8.0 kb [1,2]. HPV has more than 230 genotypes [3], and is transmitted through sexual or skin-to-skin contact; up to 80% of sexually active individuals may be infected during their lifetime, usually without symptoms [4,5]. This virus is responsible for around 5% of all cancers; both women and men develop HPV-related cancer annually, and women with HIV are more susceptible to developing cancer [6]. Infection of the skin squamous epithelium and the mucous membranes of the upper digestive tract, respiratory tract, and genital tract can cause genital warts, laryngeal papilloma, and certain types of cancer [7,8]. Among HPV genotypes, the high-risk (HR-HPV) genotypes are associated with persistent infections that may contribute to the development and progression of cancers. Cervical cancer is the fourth leading cause of cancer mortality and the most prevalent gynaecological malignancy in women globally. The World Health Organisation (WHO) estimates that approximately 625,600 women and 69,400 men develop HPV-related cancer annually [9]. Most of these cases occur in low- and middle-income countries due to limited screening, lack of mass vaccination programs, sociocultural changes, and living in resource-limited regions. Furthermore, these genotypes account for nearly 60% of oropharyngeal, penile, and head and neck cancers, and also for more than 90% of anal and cervical cancers [3].

Meanwhile, low-risk (LR-HPV) genotypes induce benign growth of infected cells and commonly develop warts [10]. Most HPV lesions, whether high- or low-risk, are usually self-limited. The majority of the recognised genotypes generate benign hyper-proliferative lesions and do not frequently lead to cancer in the general population. The HR-HPV genotypes, 16, 18, 31, 33, 35, 39, 45, 51, 52, 56, 58, 59, and 66, cause malignancy [11,12]. HPV types 16, 18, 31, 33, and 45 are among the most common high-risk genotypes associated with cervical cancers [13]. The WHO Cervical Cancer Elimination Modelling Consortium (CCEMC) proposed three strategies for HPV elimination: vaccination, screening, and treatment of cervical cancer [14]. WHO has strong confidence that implementing these strategies not only considerably reduces HPV infections but also diminishes cervical cancer and can save millions of women’s lives in over ~80 developing countries [15]. However, for such a program, the reliable HPV method for diagnosis, genotyping, and epidemiological screening is the first step. Furthermore, the epidemiologic studies suggested that the frequency of single or multiple HPV in a single subject was rising, particularly in developing countries [16–18].

Therefore, this study was conducted in a tourism-influenced region in northeast Iran, with ~8 million inhabitants and more than 25 million pilgrims and tourists annually in North and Razavi Khorasan. However, South Khorasan is a desert land and drought-stricken area with a traditionally oriented population (https://irandataportal.syr.edu/census/census-2016). Khorasan refers to three provinces, North, Razavi, and South; previously, we performed a molecular epidemiological study eight years ago during 2013–2018 [19], and now compared the changes during eight-year intervals in HPV prevalence and genotypes in such regions.

## Materials and methods

### Ethics and study settings

This study was performed in accordance with the principles of the Declaration of Helsinki. The study was reviewed and approved by the Biomedical Research Ethics Committee of the Mashhad University of Medical Sciences **[IR.MUMS.IRH.REC.1402.178]**. All the participants obtained and signed the written informed consent forms. For minors, informed consent was obtained from a parent and/or legal guardian for participation in the study. All methods were performed in accordance with relevant guidelines and regulations.

In 2021, our first study on monitoring HPV genotype prevalence in three areas from 2013 to 2018 was reported [19]. The present study was conducted to evaluate dynamic changes in HPV from 2018 to the end of 2024 (an approximately 8-year interval). Therefore, the samples were accessed on January 25, 2024. This cross-sectional laboratory-based study was performed at molecular diagnostic laboratories affiliated with Mashhad University of Medical Sciences (MUMS) in northeastern Iran. Building on our previous work, the present study was designed to evaluate temporal changes in HPV prevalence and genotype distribution from 2018 onwards. For this purpose, HPV testing records and corresponding clinical data were retrieved up to January 25, 2024.

The study population consisted of 2,673 individuals (2,121 women and 552 men) with clinical suspicion of sexually transmitted infection (STI), referred for HPV testing from the three provinces of Khorasan (North, Razavi, and South Khorasan), as well as a small number of non-resident tourists temporarily present in these provinces. The subjects represented diverse socioeconomic backgrounds, ages, and genders. Inclusion criteria included physical examination, the presence of inflammation, no history of substance abuse, and no history of incarceration. The eligible subjects were referred to the molecular sections of referral diagnostic laboratories by specialist physicians, mostly gynaecologists, dermatologists, infectious disease specialists, and urologists, for HPV DNA testing and genotyping.

### Sample collections

Patients were admitted to the molecular laboratories under the supervision of Mashhad University of Medical Sciences. Expert midwives collected cervical specimens from female participants using liquid-based cytology (LBC kit; Viva Cell, Iran) according to the manufacturer’s manual. Solid tissue samples were collected by a gynaecologist and fixed in formalin. The laboratory prepared paraffin-embedded samples, and six 4 µm sections were used for DNA extraction.

### DNA extraction

Immediately after sample collection, HPV DNA specimens were extracted using a DNA extraction kit (Roche, Germany) according to the manufacturer’s instructions. DNA was stored at −20 °C until further analysis.

### HPV detection and genotyping

HPV genotyping was performed using the PCR-hybridisation method with the High+Low PapillomaStrip® kit (OPERON S.A. Immunodiagnostics, Spain) according to the manufacturer’s instructions. The kit detects 37 different low- and high-risk HPV types (6, 11, 16, 18, 26, 31, 33, 35, 39, 40, 42, 43, 44, 45, 51, 52, 53, 54, 56, 58, 59, 61, 62, 66, 67, 68, 69, 70, 71, 72, 73, 74, 81, 82, 83, 84 and 91).

### Statistical analyses

SPSS version 11.5 (SPSS, Chicago, IL, USA) was used to analyse data. The descriptive statistical analyses, including central and dispersion indices, are presented in tables. Due to the nature of the variables, descriptive results were analysed using a non-parametric *Chi*-square test. A *p*-value less than 0.05 was considered statistically significant.

## Results

### HPV in the studied population

The study included 2,673 referred participants, aged 11–75 years, who had vaginal or urethral inflammation suspected of STI. Of 2,673 STI-suspected subjects, 2,121 (79.34%) were female and 552 (20.66%) male. PCR detected HPV DNA in 1,185 (55.86%) females and 400 (72.47%) males. On the other hand, 1,088 were negative, including 939 (44.24%) females and 152 (27.53%) males (Table 1). Generally, HPV infection prevalence was significantly higher among males than among females (*p* = 0.01).

**Table 1:** The frequency of HPV-positive and negative subjects according to gender.

Subjects distribution		Gender	No. (%)		mean±SD
Local population	North and Razavi Khorasan	Female	Negative	745 (39.95)	34.7 ± 9.05
			Positive	1120 (60.05)	33.0 ± 8.98
		Male	Negative	145 (26.9)	32.0 ± 7.49
			Positive	394 (73.1)	32.4 ± 8.68
	South Khorasan	Female	Negative	130 (80.75)	33.4 ± 7.13
			Positive	31 (19.25)	30.5 ± 6.56
		Male	Negative	2 (100)	31.5 ± 2.12
			Positive	0 (0%)	–
	Tourists	Female	Negative	61 (64.21)	39.5 ± 10.64
			Positive	34 (35.79)	34.6 ± 8.89
		Male	Negative	5 (45.45)	47.4 ± 14.31
			Positive	6 (54.55)	37.5 ± 6.57

The age of the study population was 34.54 ± 8.9 and 32.84 ± 8.8 in the HPV-negative and HPV-positive groups, respectively. The mean age of females was 34.78 ± 9.04 and 33.02 ± 8.8 in the HPV-negative and HPV-positive groups, respectively. Moreover, the mean age of males was 32.8 ± 8.47 and 32.21 ± 8.87 in the HPV-negative and HPV-positive groups, respectively.

The participants were divided into seven age groups, and, as shown in Table 2, the lowest infection rate was observed in those >60 years old (0.7%). However, HPV infection was mainly frequent in sexually active age groups, 21–30 and 31–40 years old groups, 35.6% and 39.7%, respectively (Table 2). The ratio of HPV-positive females to males in the 31–40 age group was 838/222 (3.7), *i.e*., 3.7 times higher in females than in males. Further, STI-suspected subjects with HPV infection, 42.5% (674/1585), had a single infection, 26.6% (422/1585) two genotypes, 14.4% (229/1585) three genotypes, 7.8% (124/1585) four, and 8.6% (136/1585) more than four genotypes (Table 2). Notably, some individuals harboured more than 11 genotypes.

**Table 2:** The frequency of HPV infection by age group and genotype.

Character	Female		Male		Total	
Age group	Frequency	Percent	Frequency	Percent	Frequency	Percent
<20	91	% 4.3	17	% 3.1	108	% 4.0
21-30	756	% 35.6	241	% 43.7	997	% 37.3
31-40	838	% 39.5	222	% 40.2	1060	% 39.7
41-50	328	% 15.5	52	% 9.4	380	% 14.2
51-60	89	% 4.2	14	% 2.5	103	% 3.9
61-70	12	% 0.6	5	% 0.9	17	% 0.6
71-75	3	% 0.1	1	% 0.2	4	%0.1
Genotypes	**Female**		**Male**		**Total**	
Negative	936	% 44.1	152	% 27.5	1088	% 40.7
Single	527	% 24.8	147	% 26.6	674	% 25.2
2 genotypes	326	% 15.4	96	% 17.4	422	% 15.8
3 genotypes	161	% 4.6	68	% 12.3	229	% 8.6
4 genotypes	84	% 4.0	40	% 7.2	124	% 4.6
>4 genotypes	87	% 4.1	49	% 8.9	136	% 5.1

### Age-specific HPV positivity and trends

**Fig 1:**
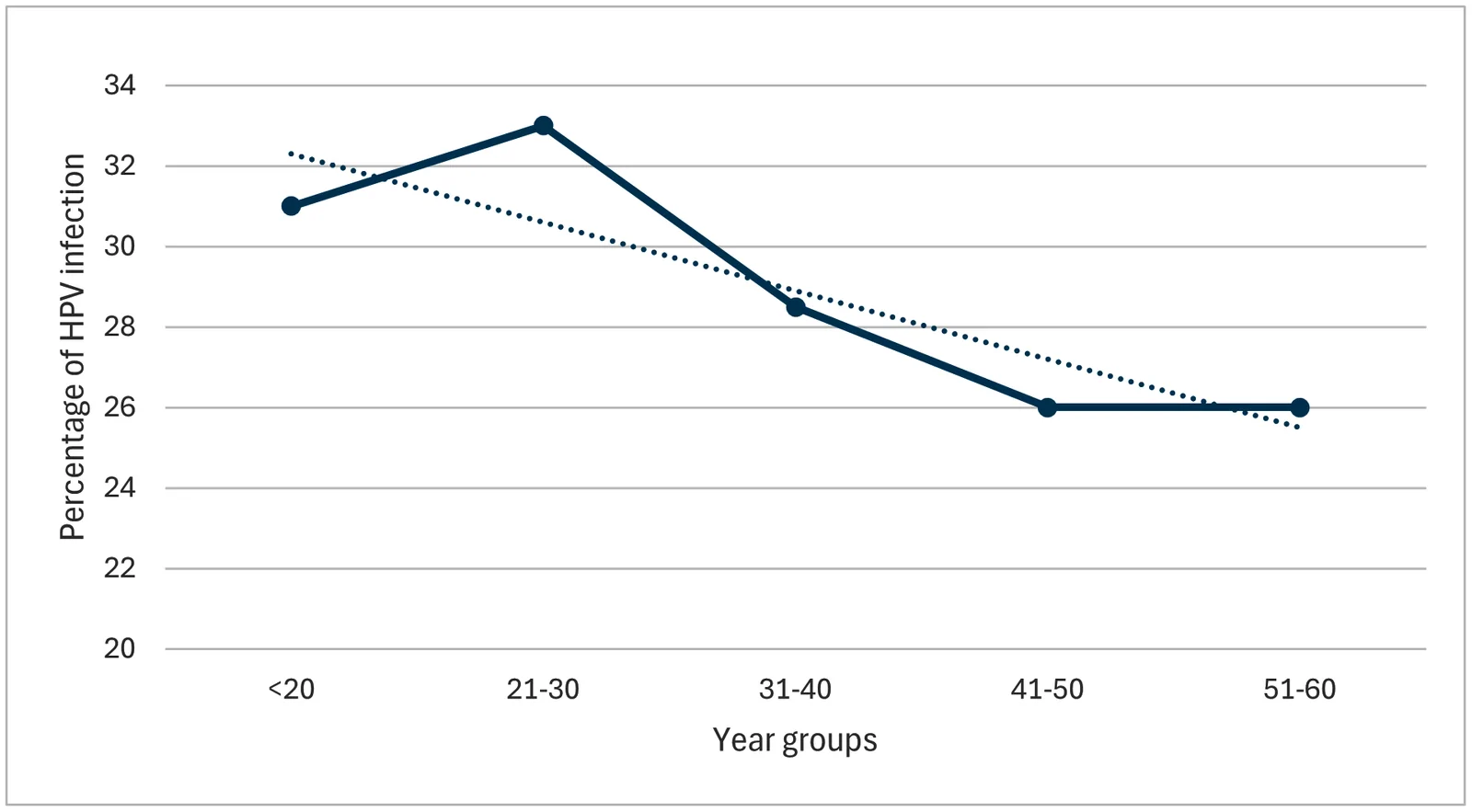
Age-specific prevalence of HPV infection in the multi-year dataset. The solid line shows the proportion of HPV-positive tests in each age group, and the dotted line indicates the fitted linear trend. Prevalence peaks in the 21–30-year group and declines progressively with increasing age.

Age-stratified analysis showed a peak in HPV positivity at 21–30 years, followed by a steady decline with increasing age. Specifically, HPV positivity (%) by age group was: < 20, 31%; 21–30, 33%; 31–40, 28.5%; 41–50, 26%; and 51–60, 26%. The fitted trend across 21–60 years was negative, consistent with decreasing prevalence beyond young adulthood (Fig 1). Age groups >60 years were excluded from trend modelling and visualisation due to small sample sizes and unstable effect estimates, which can bias slope estimation and confidence bounds.

### Age-specific distribution of multiple HPV genotype infections

**Fig 2:**
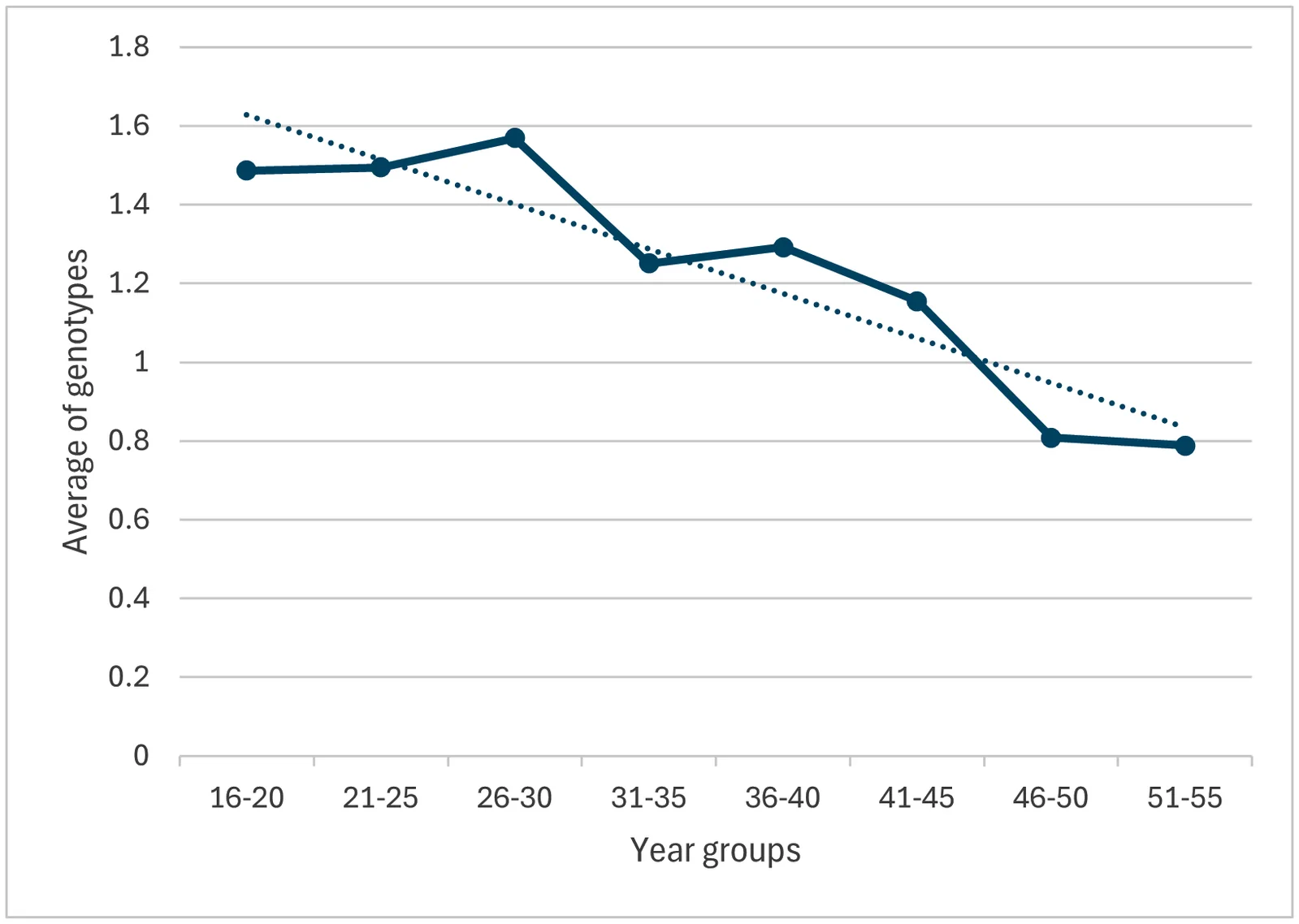
Mean number of HPV genotypes per HPV-positive sample by age group over the study period. The solid line represents the average count of distinct genotypes, and the dotted line shows the linear trend. Younger age groups have more multiple infections, with an apparent decline in the number of genotypes with increasing age.

Using all participants (HPV-negative counted as 0; each detected genotype counted as 1, *e.g*., 6 + 16 + 18 = 3), the mean number of concurrent HPV genotypes per individual was highest in young adulthood and declined with increasing age. Age-group means were 1.49 (16–20), 1.52 (21–25), 1.58 (26–30; peak), 1.27 (31–35), 1.31 (36–40), 1.16 (41–45), 0.80 (46–50), and 0.78 (51–55). Overall, the age-specific pattern indicates a progressive reduction in multiple HPV genotype infection with increasing age, with a minor secondary increase observed in the 36–40 age group (Fig 2).

### High-multiplicity HPV infection (>4 genotypes)

**Fig 3:**
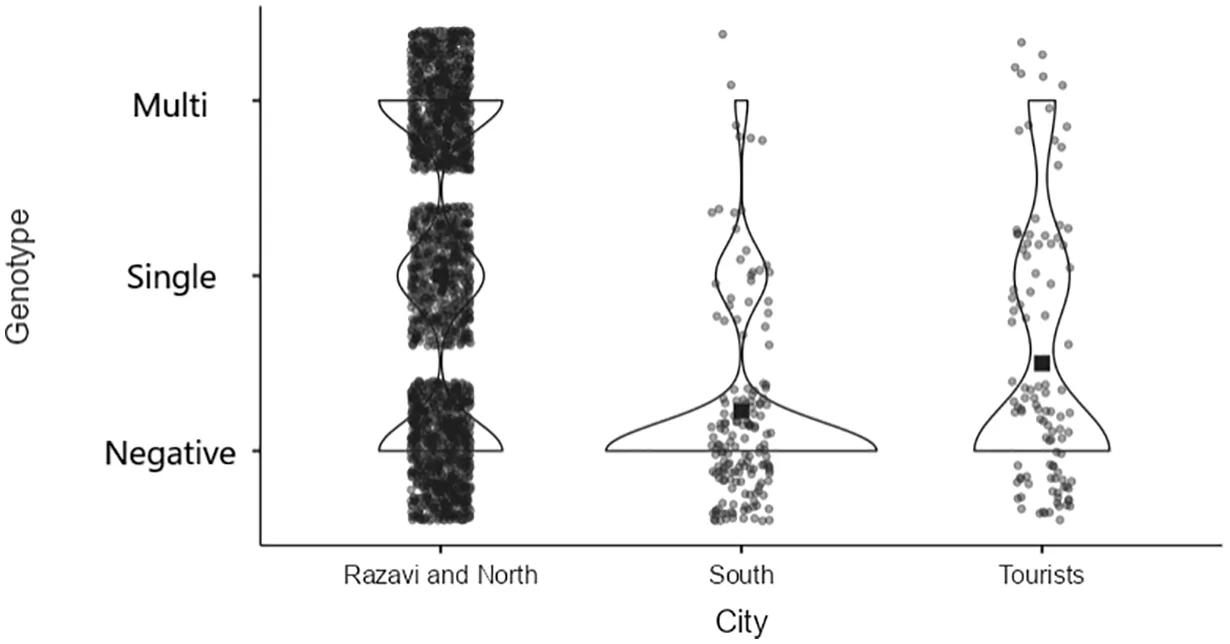
Distribution of HPV infection status (negative, single genotype, multiple genotypes) across three city groups (Razavi and North, South, and tourists) over the study period. Violin plots show the density of observations, and individual dots represent participants. The plot highlights a higher proportion of HPV-negative results in the South, and relatively more single and multiple infections in Razavi and North, as well as among tourists. Comparing the distribution of HPV single- and multi-geotypes in North and Razavi shows that temporal residence can increase the spread of HPV, while South Khorasan remains intact.

Among individuals with >4 concurrent HPV genotypes (*n* = 136), multiplicity was: five genotypes (57/136; 41.9%), six (39/136; 28.7%), seven (18/136; 13.2%), eight (12/136; 8.8%), nine (3/136; 2.2%), ten (2/136; 1.5%), 11 (4/136; 2.9%), and 12 (1/136; 0.7%), indicating that most cases clustered at five–six genotypes. The mean age was 30.9 ± 7.04 years (range 17–56), consistent with concentration in early adulthood. To preserve clarity, per-case genotype constellations are omitted from the main tables; aggregate patterns are presented in Fig 3. Genotype presence across the > 4 genotype cases (*n* = 136) showed enrichment for both LR- and HR-HPV. The six most frequent were HPV-6 (56.62%; 77/136), HPV-16 (40.44%; 55/136), HPV-54 (36.03%; 49/136), HPV-43 (29.41%; 40/136), HPV-51 (29.41%; 40/136), and HPV-52 (28.68%; 39/136). Importantly, three of these 16, 51, and 52 are classified as HR-HPV, underscoring the substantial HR-HPV burden within multi-genotype infections rather than a pattern driven solely by LR-HPV (Fig 3).

Table 3 shows the distribution of sample types, including paraffin-embedded tissue, vaginal discharge, fresh solid tissue, and urethral discharge.

**Table 3:** The type of referral samples for HPV PCR and genotyping.

Sample type	Female		Male		Positive	
Paraffin-embedded tissue	140	% 6.6	3	% 0.5	29	% 1.8
Genital discharge	1941	% 91.5	9	% 1.6	1133	% 71.5
Fresh solid tissue	1	0	5	% 0.9	2	% 0.1
Urethral discharge	0	0	532	% 99.5	393	% 24.5

In the present study, 34 HPV genotypes (HR and LR) were identified by PCR-hybridisation methods. The highest prevalence of HPV genotypes was genotype 6 (36.4%), followed by genotypes 16 (17.03%), 51 (12.49%), 54 (12.11%), 42 (10.28%), 39 (10.09%), 56 (8.76%), and 44 (8.20%) (Table 4).

**Table 4:** The frequency of each HPV genotype in the studied population.

HPV genotype	Frequency	Percent (%)
6	578	36.4%
16	270	17.3%
51	198	12.49%
54	192	12.11%
42	163	10.28%
39	160	10.09%
56	139	8.76%
44	130	8.20%
66	124	7.82%
52	124	7.82%
11	119	7.50%
43	104	6.56%
31	99	6.24%
53	95	5.99%
40	90	5.67%
68	82	5.17%
62	77	4.85%
81	74	4.66%
45	70	4.41%
73	65	4.10%
82	64	4.03%
58	64	4.03%
59	63	3.97%
91	62	3.91%
18	60	3.78%
67	57	3.59%
35	55	3.47%
55	51	3.21%
84	23	1.45%
74	19	1.19%
83	13	0.82%
89	11	0.69%
33	10	0.63%
70	6	0.37%

The results revealed that the most common genotype in this study was HPV-6, with a frequency of 36.4%, followed by HPV-16, with a frequency of 17.03%. Table 4 lists the frequencies and prevalence of the different genotypes.

### Adolescent subgroup (11–18 years)

Although the number of participants aged 11–18 years (*n* = 38) was low, HPV positivity was 50.0% (19/38). In terms of gender, 3/8 males were positive (37.5%), and 16/30 females were positive (53.3%). Genotype distribution in this age band was dominated by HPV-6, detected in 11 cases (57.9% of positives). HPV-11 was identified in three cases (15.8%), and HPV-16, HPV-42, and HPV-54 were identified in two cases (10.5% each). Multi-genotype infections were common: 12 (63.2%) cases had single-genotype infections, 4 (21.1%) harboured two genotypes, and 1 (5.3%) case carried three genotypes.

### Changes in HPV prevalence and the number of genotypes after an eight-year interval study

Overall, in the studied population, HPV prevalence has risen by more than 28% compared to eight years ago. The most significant change in the number of genotypes per person was > 4, which is more than 12 times higher than 8 years ago (0.4% *vs* 5.1%). Also, less change was observed in subjects with three genotypes in one subject (Table 5). The most significant changes in HPV prevalence occurred in tourist provinces, increasing by around 14.5%, followed by a diverse group at 3.2%. However, among traditionally oriented people in South Khorasan, the density of 5.1 individuals/km2 remained unchanged, even though the infection rate decreased by 0.9%.

**Table 5:** Change in HPV positivity over eight years in the studied populations.

HPV infection and genotypes	Previous study (%)	Present study (%)	Changes (%)
Negative	64.7%	40.7%	**− 24%**
Positive			
Single	13.9%	% 25.2	**+11.3%**
2 genotypes	11.2%	% 15.8	**+4.6%**
3 genotypes	4.8%	% 8.6	**+3.8%**
4 genotypes	0.4%	% 4.6	**+ 4.2%**
> 4 genotypes	0.4%	% 5.1	**+4.7%**
HPV positivity changes over eight years.			**+28.6%**

### Yearly trend in HPV genotype-specific prevalence (2015–2023)

**Fig 4:**
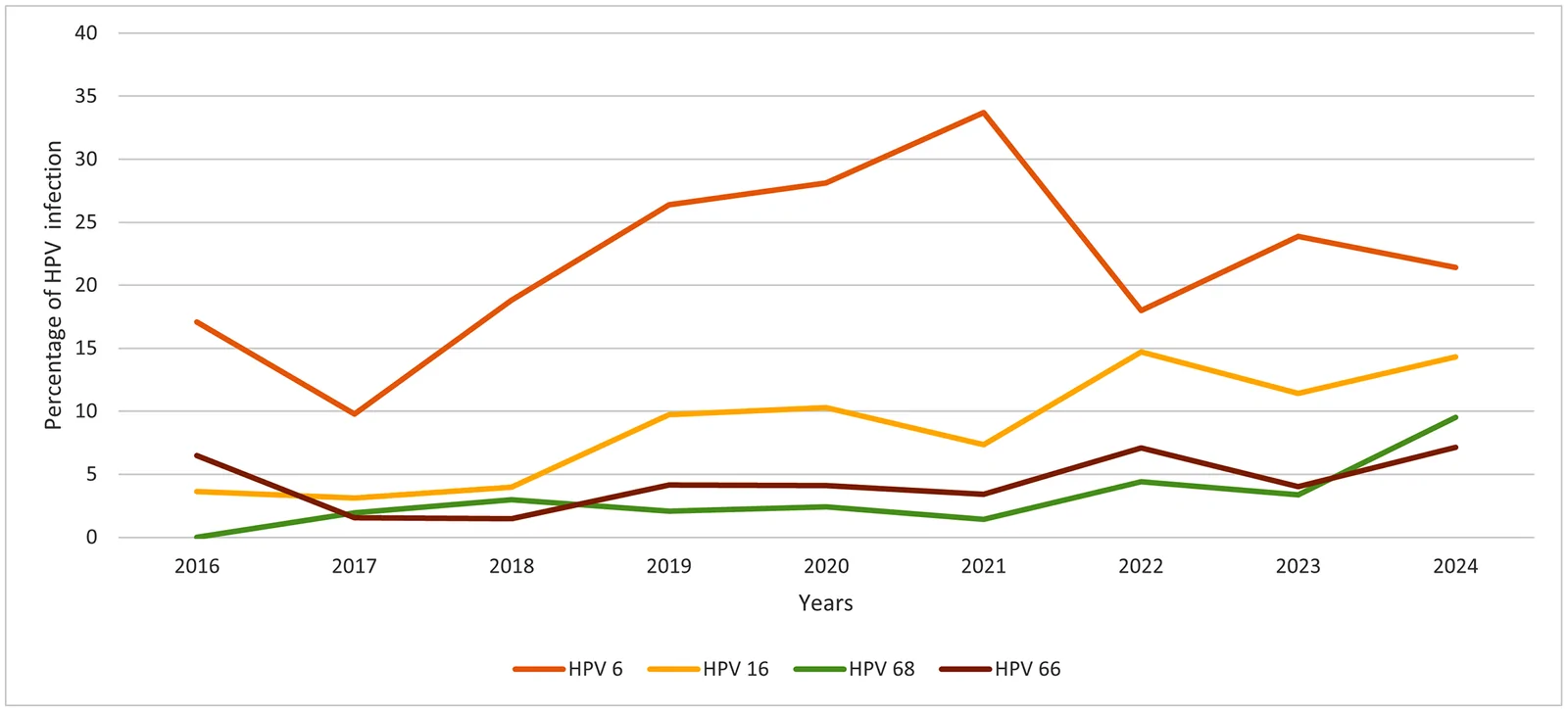
Temporal trends in the prevalence of HPV genotypes 6, 16, 68, and 66 from 2016 to 2024. Solid lines display annual percentages for each genotype. HPV 6 remains the most frequent type, while all four genotypes demonstrate overall increasing trends despite year-to-year fluctuations.

Using aggregated data from our referral labs, past and present annually, we plotted temporal trends for four representative HPV genotypes spanning the International Agency for Research on Cancer (IARC) risk categories: HPV-6 (low-risk), HPV-16 (high-risk), and the possible and probably oncogenic types HPV-66 and HPV-68. HPV-6 showed a non-monotonic pattern with a pronounced peak in 2020 (33.7%), followed by a decline and partial stabilisation (23.9% in 2022; 21.4% in 2023), yielding a modest net increase versus 2015 (+4.3 percentage points; + 25.1% relative to baseline) Table 6. HPV-16 rose substantially over the period, from 3.65% (2015) to 14.3% (2023), with step-ups around 2018–2019 and again in 2021 (peak 14.7%), indicating a marked upward drift despite year-to-year variability. HPV-68, initially near zero (2015), increased gradually with a notable uptick in 2023 (9.52%), while HPV-66 fluctuated (low in 2016–2017), then rebounded to 7.14% by 2023 (from 6.5% in 2015). The annual trends in HPV-dominant genotypes are depicted in Fig 4.

**Table 6:** Changing level of HPV positivity during an eight-year interval according to the locations (provinces).

Provinces	Study time	Negative (%)	Positive (%)	Changes (eight-year interval (%))
North and Razavi Khorasan	Past	51.6%	48.4%	14.5%
	Present	37.1%	62.9%	
South Khorasan	Past	80.1%	19.9%	0.9%
	Present	81.0%	19.0%	
*Diverse group	Past	66.4%	33.6%	3.2%
	Present	63.2%	36.8%	

*Diverse group: Out of Khorasan provinces (North, Razavi, and South).

### Geographic pattern of HPV infection status

The distribution of HPV infection status differed markedly between the three geographic groups (Fig 3). In Razavi and North Khorasan, the density of observations was concentrated among single- and multi-genotype infections, indicating that most residents harboured at least one HPV genotype and that multi-genotype infections were common. In contrast, samples from South Khorasan clustered predominantly in the “negative” category, with relatively few single-type and rare multi-type infections, consistent with the lower overall HPV prevalence observed in this province. The pattern in the tourist group closely resembled that of Razavi and North Khorasan, with a clear shift toward single and multi-genotype infections and a smaller proportion of HPV-negative individuals, suggesting that tourists contribute to a high-burden profile comparable to that seen in the main pilgrimage province.

## Discussion

HPV distribution varies across countries and within countries. The malignancy burden attributed to HPV is strongly associated with income level, density, and health system condition [20,21].

Compared with our previous study published in 2021, overall HPV prevalence is higher in North and Razavi Khorasan and in the diverse group, whereas it remains stable in South Khorasan. Overall, HPV positivity in the current survey was higher than it was 8 years ago. HPV-6 dominated the genotype profile as the leading low-risk type, followed by HPV-16 as the most common high-risk type, with HPV-51 also contributing substantially to the burden.

The most common genotypes associated with an HR of cervical cancer were genotype 51 in the first group and genotype 16 in the second and diverse groups. Among the LR genotypes, HPV-6 and HPV-11 were more common in all groups. In the first group, 11.2% had two genotypes, 4.8% had three genotypes, and 0.4% had four or more genotypes [19].

Overall, HPV genotypes were detected in 1,585 patients (59.29%), an increase in prevalence of HPV compared to the previous study. The prevalence of common genotypes was similar to that reported in the previous study, with genotype 6 being the most common LR (36.4%), and genotypes 16 and 51 were the most common HR genotypes, each identified in 17.3% and 12.49%, respectively.

As Fig 3 shows, the pattern suggests that high population mobility driven by pilgrimage and tourism, along with population density in Razavi and North Khorasan, may be among the factors promoting HPV transmission and the accumulation of multi-type infections. In contrast, the more traditional-oriented, low-mobility context and population density (5.1 individuals/km^2^) of South Khorasan are associated with lower HPV burden and fewer complex infections. Therefore, it can be speculated that population density, migration and tourism in these provinces profoundly affect the epidemiology of chronic infectious diseases such as HPV (Fig 3). Furthermore, the study showed that age, gender, and traditionally oriented (conservative) behaviours seem to affect HPV as a chronic infection (Figs 1–3).

The observed peak in HPV positivity at 21–30 years aligns with the typical window of highest social activities and contacts. After this, cumulative immunity, behavioural changes, and sociocultural stabilisation likely reduce acquisition risk and the detectability of infections. The gradual decline from 31–40 onward mirrors patterns reported in many STI surveillance cohorts, in which incident HPV infections decline with age. In contrast, persistent high-risk infections constitute a smaller fraction of positives (Fig 1). Taken together, these data support prioritising vaccination before sexual debut, sustaining coverage through early adulthood, and maintaining screening in later decades to capture persistent high-risk infections rather than incident acquisitions [22,23].

The concentration of higher genotype burden in young adulthood is consistent with the peak of social activities and contacts, which increases exposure opportunities and co-acquisition of multiple HPV types. The progressive decline from the mid-30s onward likely reflects a combination of sociocultural stabilisation, partial type-specific immunity, and enhanced clearance of incident infections with age, leaving fewer concurrent detectable genotypes [18]. The small secondary rise at 36–40 may reflect cohort or referral effects rather than a sustained pattern (Fig 2).

Methodologically, treating negatives as zero provides an intention-to-survey estimate of population-genotype burden rather than restricting to HPV-positive individuals, thereby avoiding upward bias in older age bands where positivity is lower. Collectively, these data support prioritising vaccinations before and through early adulthood and maintaining screening at older ages, with a focus on the persistence of high-risk infections rather than on multigenotype acquisition.

In the present study, the most common LR genotype was genotype 6, as in the previous study, and the most common HR genotype was genotype 16, in contrast to the previous study. Notably, HPV genotype prevalence increased dramatically across the entire study population. Unexpectedly, a greater increment was observed in the category of more than 4 genotypes per subject, which was 11.7 times higher than in the previous study; the increase was observed in Razavi Khorasan, whereas in traditional-oriented South Khorasan, it was rare. The HPV prevalence rate was higher than in a study conducted by Kesheh *et al.* on 8,351 subjects in 2019, and the HPV prevalence rate in the Iranian population was estimated at 42%. Similarly, genotype 6 was the most common genotype [24]. This indicates the uniformity of the geographical distribution of genotype 6 in a country. In another study in Mashhad 2017, on 143 cervical cytology samples in which the presence of HPV was confirmed, contrary to our study, the most common genotype was from the HR group (genotype 16 with a frequency of 38.6% and genotype 18 with 14.7%), which could be due to the differences in the methodology and the size and form of studied population, which consisted of people with low-grade squamous intraepithelial lesions [25].

Since genotypes 16 and 18 are HR groups, they are likely common among people with precancerous lesions. Moreover, a study on the prevalence of HPV in Iranian women from the general population living in 11 provinces of Iran is estimated to be at 2.4% (108 out of 2,562 people). The highest prevalence of HPV was in the age group of 25–34 years, which is consistent with our study. The prevalence of HR/ intermediate-risk and LR HPV genotypes was estimated at 3% and 1.2% of the total female population, respectively. Additionally, the highest prevalence was related to genotype 16 [26].

In the > 4 genotype subgroup, three of the six most frequent types were HR-HPV (16, 51, 52, per your lab’s schema), indicating that multigenotype infections in the present study are not driven solely by LR-HPV but carry a substantial oncogenic burden. This pattern is consistent with reports showing that multiple HPV infections—particularly those involving HR-HPV—are more common in higher-grade cytology/histology and that HR-HPV beyond HPV-16 (e.g., 51/52/58) are over-represented when multiple types co-occur (Fig 3). In terms of pathogenesis, concurrent acquisition from overlapping partnerships, type-specific differences in persistence/clearance, and assay sensitivity to mixed infections likely contribute to this enrichment; clinically, the co-presence of several HR-HPV types may increase the probability that at least one persists and drives lesion progression, warranting vigilant follow-up and vaccination strategies covering broader HR-HPV spectra [27].

A study at the University of Oklahoma on 1,670 women found a very high prevalence of multiple HPV infections with up to 14 genotypes detected in single specimens. Genotype 16 showed a significant trend toward more infections at a younger age [18]. There were consistencies with our present study. In another study by Maria *et al*., conducted in southern Italy in 2015, multiple infections with two different HR-HPV genotypes were reported in 10% of samples [28]. The findings showed that the prevalence of multi-genotype infections in the population studied has increased significantly compared to a few years ago. Although the most common LR did not change, for HR-genotype, genotype 51 in the previous study is now genotype 16. Health authorities should focus more on establishing HPV diagnostic facilities, expanding screening and vaccination, and increasing public knowledge in these areas. In Muslim countries, particularly Iran, extramarital affairs are strictly prohibited. Therefore, other modes of transmission should also be investigated, such as skin-to-skin contact, mother-to-child transmission during delivery, shared toilets, swimming pools, large water bodies, and autoinoculation from a viral wart [29]. The eligible subjects in this study were the resident population from all socioeconomic groups and were randomly referred by gynaecologists, urologists, or dermatologists for presumptive HPV infection. Therefore, as a tourist and pilgrimage region attracting more than 30 million mobile visitors each year from other Iranian provinces, the Middle East, and Central Asia, along with some economic problems, changes are expected. However, infectious disease surveillance is particularly important in such an area to monitor associated disease burden, such as cancers in females and males, and to recognise outbreaks. The authors observed such conditions for HEV in this region [30].

Although in the underage subgroup, the positivity was around 50%, the main predominance genotype was low-risk HPV-6, and a non-trivial rate of multigenotypic infections. Considering the low-risk genotypes, possible transmission routes, such as vertical/perinatal transmission, non-sexual transmission, and autoinoculation, were inaccessible. It can be speculated that the prevalence of warts in this group. Overall, the results in this group reinforce the public-health value of timely HPV vaccination prior to sexual debut, sustained coverage through adolescence, and continued molecular surveillance that captures both genotype spectrum and coinfection patterns in younger populations. Limitations include the modest sample size and referral-based enrollment, which may inflate observed prevalence relative to the general adolescent population. However, a meta-analysis on 15 studies for HPV prevalence in the population under 12 years old found 25.5% with higher positivity born to positive mothers than negative mothers [31].

Some vaccine brands are available in Iran, but not under social health insurance, and should be prescribed by professional physicians. However, in the case of chronic infection, such as HPV, the main responsibilities lie with local governors and health authorities.

Furthermore, according to the WHO ICO/IARC Information Centre on HPV and Cancer factsheet (2023) (https://hpvcentre.net/statistics/reports/IRN_FS.pdf), in Iran, around 33.5 million people are aged more than 15 and at risk of developing cervical cancer, which is mainly related to the HPV-16 and 18 genotypes. Furthermore, according to the more reliable report of the National Cancer Registry of Iran (Ir. NCR), during these years, cervical cancer ranked 11^th^ and 12^th^ among all cancers in Razavi Khorasan and North Khorasan, respectively (Table 7). Meanwhile, the national data also had a relatively stable trend. The documented Ir-NCR initiated in 2017 also reported the incidence of cervical cancers in the country and provinces. However, the data in the first year may be biased by a large-scale cervical cancer screening campaign in the region. The five-year data has still not been released.

**Table 7:** The report of The National Cancer Registry of Iran (Ir. NCR) for cervical cancer in Khorasan provinces, North, Razavi, and South, in 2018 and 2019.

Locations	Years	Number	Crude/10^5^	ASR/10^5^	Rank among women	Rank in all
Razavi Khorasan	1397	169	5.17	5.23	11	12
	1398	169	4.26	4.35	11	12
North Khorasan	1397	22	5.16	5.55	8	12
	1398	25	5.21	5.61	7	11
South Khorasan	1397	15	3.76	3.51	11	–
	1398	13	3.62	3.21	12	–
Iran	1397	2621	6.5	6.47	7	11
	1398	2622	6.5	6.47	7	11

Age-standardised rate: ASR.

The trends in HPV prevalence and burden of cervical cancers, health authorities and disease control and prevention centers need to harness the potential of social media to meet the information needs of youth and to provide education and awareness, especially regarding HPV transmission and prevention [32].

The present study has some limitations; I- it is possible to obtain smear pathology for comparison with molecular methods. II- the possible transmission routes, including vertical/perinatal transmission, non-sexual transmission, autoinoculation, and skin wart contamination, could be considered. III- a standard questionnaire may help determine socioeconomic status, classification by living district, and educational levels. IV- It was important to have behavioural or sexual-history data. Finally, due to cultural practices in these provinces, access to some of this data could be difficult.

## Conclusion

After eight years of work and widespread media coverage on HPV knowledge, no significant improvement has occurred in this chronic public health issue. Our findings revealed that Razavi Khorasan, as a pilgrimage and tourist province, showed a sharp upward trend in HPV prevalence as a chronic infection, particularly multi-genotypes, which are spreading within the resident population over eight-year intervals. The upward trend in HPV prevalence underscores the importance of vaccination against HPV, particularly for children under 15 years old, and increasing awareness of the virus as an STI for early detection. Therefore, the health authorities in the North and Razavi Khorasan provinces must consider education, monitoring, planning, and action efforts to prevent HPV from spreading further. Monitoring and screening for prostate cancer in males and cervical cancer in females is crucial for HPV-infected subjects. It is more effective to incorporate health education into social media to achieve the HPV screening coverage outlined above.

## Supporting information

S1 Fig The graphical representation of the HPV infection by regional distribution. (TIF)

S2 Fig The frequency and percentage of major HPV genotypes, with sex-specific distributions. (TIF)
